# E3 ubiquitin ligases in bone homeostasis: from regulatory mechanisms to skeletal diseases and therapeutic targeting

**DOI:** 10.3389/fcell.2026.1825526

**Published:** 2026-05-18

**Authors:** Yutong Zhao, Nanjue Cao, Wenxiao Yang, Yuanfang Wang, Wei Wang

**Affiliations:** Liaoning Provincial Key Laboratory of Oral Diseases, School and Hospital of Stomatology, China Medical University, Shenyang, China

**Keywords:** bone homeostasis, E3 ubiquitin ligases, molecular mechanisms, osteogenesis, ubiquitin–proteasome system

## Abstract

E3 ubiquitin ligases are key determinants of substrate specificity within the ubiquitin–proteasome system and have emerged as important regulators of skeletal biology. Although traditionally classified into RING-, HECT-, and RBR-type families based on structural features, their roles in bone homeostasis are more clearly understood when examined in the context of specific skeletal regulatory processes. In this review, we briefly outline major E3 ligase families and the updated human E3 ligase landscape, and then discuss how representative E3 ligases regulate osteoblast differentiation, osteoclast differentiation, cartilage homeostasis, and bone remodeling. A central concept emerging from current evidence is that E3 ligases converge on critical regulatory nodes that govern skeletal cell fate and tissue-level remodeling. In osteogenesis, multiple E3 ligases control RUNX2 stability, transcriptional activity, and associated signaling pathways, including the BMP/SMAD and PI3K/Akt pathways. In osteoclast differentiation, E3 ligases primarily modulate TRAF6-dependent signaling and NF-κB activation, thereby influencing bone resorption. Beyond these lineage-specific roles, E3 ligases also participate in higher-order processes such as osteoblast–osteoclast coupling, osteoimmune regulation, mitochondrial quality control, and cartilage matrix homeostasis, highlighting their roles as process-level regulators of skeletal homeostasis. We further summarize how dysregulation of E3 ligase–mediated pathways contributes to common skeletal disorders, including osteoporosis, inflammatory bone loss, degenerative diseases, and therapy-associated complications. Finally, we discuss emerging pharmacological strategies targeting E3 ligases and E3-dependent pathways, including modulation of ubiquitination signaling and targeted protein degradation. Collectively, this review underscores the central roles of E3 ubiquitin ligases in integrating skeletal regulation and highlights their potential as therapeutic targets in bone-related diseases.

## 1 Introduction

Bone defects can result from congenital malformations, severe trauma such as fractures, malignant neoplasms, infections, including osteomyelitis and periodontitis, osteoporosis, and other skeletal disorders. Repairing these defects remains a significant clinical challenge and a major global public health concern, substantially affecting patients’ quality of life (Zhao X. et al., 2024; Li et al., 2025b).

Skeletal development, growth, and regeneration depend on precisely coordinated ossification processes. These include intramembranous ossification, in which osteoblasts originate directly from chondrocytes within the growth plate, bone marrow–derived mesenchymal stem cells, quiescent bone-lining cells on bone surfaces, and specialized craniofacial fibroblasts, as well as endochondral ossification, which proceeds via a transient cartilage template (Duchamp de Lageneste et al., 2018; Mizoguchi and Ono, 2021). Consequently, bone is a highly dynamic tissue whose structural integrity and functional competence are maintained through tightly regulated remodeling processes driven by the coordinated activity of osteoblasts and osteoclasts, ensuring overall bone homeostasis (Jin et al., 2025). Maintaining this equilibrium requires not only the interplay of cellular activities but also precise molecular regulatory networks, among which E3 ubiquitin ligases have emerged as pivotal determinants of protein turnover and signaling specificity in skeletal physiology and pathology.

Ubiquitin (Ub) is a conserved 76-amino acid protein containing seven lysine residues (K6, K11, K27, K29, K33, K48, and K63) and is expressed ubiquitously in eukaryotic cells. It can be covalently attached to substrate proteins, either to target misfolded proteins for degradation or to modulate protein stability and function (Lyu et al., 2022; Castañeda et al., 2016). As a posttranslational modification (PTM), ubiquitination determines the fate of proteins by conjugating ubiquitin to lysine residues. This modification participates in numerous biological processes, including metabolic reprogramming, DNA repair, cell cycle regulation, immune responses, and programmed cell death (Geffen et al., 2023; Wang X. et al., 2023). By fine-tuning intracellular protein homeostasis, ubiquitination contributes to organismal balance by regulating cell cycle progression, DNA repair pathways, immune signaling, and diverse physiological and pathological processes (Qiu et al., 2023; Li et al., 2021).

**FIGURE 1:**
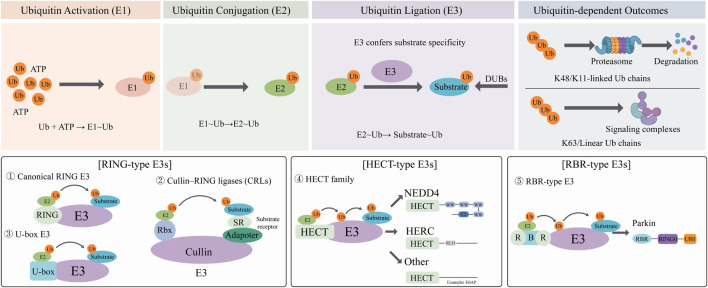
The process of ubiquitination and major classes of E3 ubiquitin ligases: Ubiquitination is initiated by E1, which uses ATP to activate ubiquitin and form an E1∼Ub thioester. Next, ubiquitin is transferred from E1 to E2, forming an E2∼Ub thioester. E3 confers the substrate specificity, recruits the E2∼Ub and the substrate, and catalyzes ubiquitin transfer. Different ubiquitin chain linkages lead to different outcomes, including proteasomal degradation (often K48- or K11-linked chains) and non-proteolytic signaling (often K63-linked or linear chains). E3 ubiquitin ligases are classified into three major families: RING-type (canonical RING, Cullin–RING ligases, and U-box), HECT-type (NEDD4, HERC, and other HECT E3s), and RBR-type E3s (e.g., Parkin).

The ubiquitin–proteasome system (UPS) constitutes the principal non-lysosomal route for intracellular protein degradation. Through a sequential enzymatic cascade involving ubiquitin-activating (E1), ubiquitin-conjugating (E2), and ubiquitin-ligating (E3) enzymes, the UPS tightly regulates protein turnover, functional modulation, and subcellular distribution (Morreale and Walden, 2016; Pao et al., 2018). Among these enzymes, E3 ubiquitin ligases confer substrate specificity by selectively recognizing target proteins and catalyzing or facilitating the transfer of ubiquitin (Ebadi et al., 2025). Ultimately, ubiquitination results in the covalent attachment of ubiquitin to lysine residues on substrates (Qiu et al., 2016). While the E1 and E2 families are relatively small, comprising 2 and 42 members respectively, the E3 family is notably expansive, with several hundred members identified to date (Deshaies and Joazeiro, 2009). Based on structural features and catalytic mechanisms, E3 ubiquitin ligases are categorized into three principal families: HECT (homologous to the E6AP carboxyl terminus), RING (really interesting new gene), and RBR (RING-between-RING) ligases (Dutta et al., 2025; Huang et al., 2024) (Figure 1).

Mounting evidence indicates that components of the UPS, particularly specific E3 ligase families, act as central modulators across a wide range of physiological and pathological contexts. By regulating epigenetic factors, core signaling molecules, epithelial–mesenchymal transition (EMT) regulators, DNA repair proteins, transcription factors, and immune checkpoint components, E3 ligases have been implicated in cancer, cardiovascular diseases, and skeletal disorders (Gao et al., 2025).

Recent advances, especially in mapping the human E3 ligase landscape (E3-ome), have refined the classification and functional annotation of E3 ligases. This framework currently identifies 672 high-confidence E3s, emphasizing their extensive functional diversity and tissue-specific roles (Chua et al., 2026). In this review, we first provide an overview of the major E3 ubiquitin ligase families within the UPS and incorporate the updated E3-ome framework to refine the classification of bone-related E3 ligases. We then discuss how E3 ligases, as representative regulators, control fundamental processes in bone homeostasis, including osteogenesis, osteoclastogenesis, cartilage homeostasis, and bone remodeling. Additionally, we summarize how dysregulation of these E3-mediated mechanisms contributes to common skeletal disorders and highlight emerging therapeutic strategies targeting E3 ligases and their associated pathways.

## 2 Classification and general features of E3 ubiquitin ligases in bone homeostasis

Ubiquitination and deubiquitination represent prevalent posttranslational modifications that govern protein stability, turnover, and cellular signaling (Zhang et al., 2025). In eukaryotic cells, the ubiquitin–proteasome system (UPS) functions as a highly selective and indispensable pathway for intracellular protein degradation, with its functional balance maintained by ubiquitin ligases (E3s) and deubiquitinating enzymes (DUBs), both of which contribute to bone regeneration and skeletal homeostasis (Pan et al., 2022). Among these regulators, E3 ubiquitin ligases serve as the principal determinants of substrate specificity, recognizing target proteins and facilitating ubiquitin transfer from E2 enzymes (Zheng and Shabek, 2017). Owing to their central role in modulating protein stability and signaling, E3 ligases have emerged as key modulators of bone homeostasis. They are broadly classified into RING-, HECT-, and RBR-type families.

The RING E3 ligase family, which includes over 600 members, represents the largest class of E3 enzymes. These ligases are characterized by RING or U-box catalytic domains, enabling the direct transfer of ubiquitin from E2 enzymes to substrates (Deshaies and Joazeiro, 2009; Buetow and Huang, 2016; Zheng and Shabek, 2017). Structurally, RING domains typically coordinate two zinc ions through conserved cysteine and histidine residues, whereas U-box domains, although structurally related, lack zinc-binding sites and rely on hydrogen bonds and salt bridges for structural stability (Ohi et al., 2003; Morreale and Walden, 2016). Representative members of this family include c-CBL, RNF4, BIRC7, and U-box ligases such as CHIP, E4B, and Prp19.

HECT (homologous to the E6AP carboxyl terminus) E3 ligases constitute the second largest class in mammals, with approximately 28 members. E6AP initially defined this family, the first identified HECT ligase mediating p53 ubiquitination (Shah and Kumar, 2021; Guo et al., 2024). Based on domain architecture, HECT ligases are divided into three subfamilies: the NEDD4 family, containing WW domains; the HERC family, characterized by RCC1-like domains (RLDs); and a subset of ligases lacking both WW and RLD domains (Sun et al., 2023; Singh et al., 2019). Distinct from RING-type ligases, HECT E3s form a transient thioester intermediate with ubiquitin via a conserved catalytic cysteine before transferring it to substrates, providing a unique catalytic mechanism (Riley et al., 2013). Representative HECT members include NEDD4-1, NEDD4L, SMURF1/2, WWP1/2, ITCH, HECW1, HECW2; large HERCs (HERC1 and HERC2) and small HERCs (HERC3–HERC6); as well as E6AP (UBE3A), HUWE1, EDD, TRIP12, and HACE1 (Xu et al., 2024; Pérez-Villegas et al., 2022; Hochrainer et al., 2005; Laine and Ronai, 2007). These ligases are involved in diverse cellular processes and are increasingly recognized as key regulators of bone tissue homeostasis.

RING-between-RING (RBR) E3 ligases utilize a hybrid catalytic mechanism that incorporates features of both RING-type and HECT-type ligases. Structurally, RBR ligases comprise three tandem domains: RING1, an in-between-RING (IBR) domain, and RING2. The RING1 domain binds the E2∼Ub conjugate, whereas RING2 contains a conserved catalytic cysteine that forms a transient thioester with ubiquitin prior to substrate transfer (Duda et al., 2013; Smit et al., 2012) (Figure 1).

These structural and mechanistic variations underpin the remarkable functional diversity of E3 ligases in skeletal regulation. Rather than acting uniformly, distinct E3 ligases exert context-dependent effects on osteogenesis, osteoclastogenesis, cartilage homeostasis, and bone remodeling by targeting specific substrates and signaling pathways. Accordingly, the following sections will discuss representative E3 ligases from the perspectives of bone homeostasis, skeletal disease dysregulation, and emerging pharmacological interventions, thereby highlighting their mechanistic and translational significance in bone-related disorders.

## 3 E3 ubiquitin ligases in bone homeostasis

**FIGURE 2:**
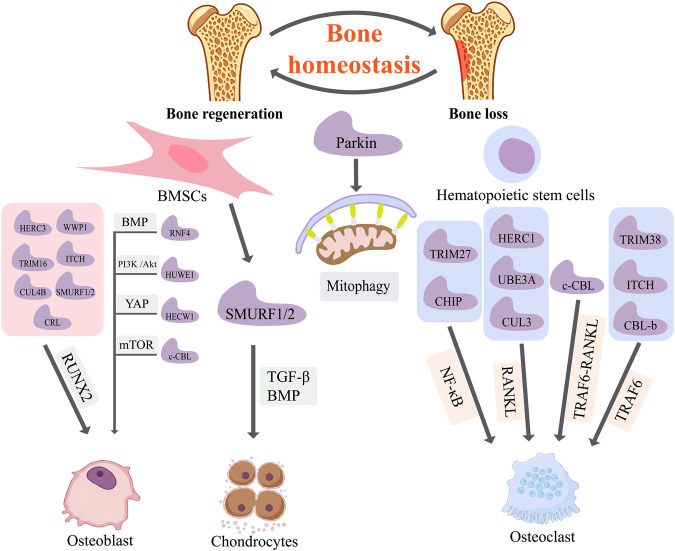
E3 ubiquitin ligases involved in bone homeostasis and their specific regulatory roles: E3 ubiquitin ligases (as indicated) regulate bone formation and resorption through osteoblast and osteoclast differentiation, as well as additional processes such as mitophagy. E3 ligases influence the differentiation of BMSCs, thereby modulating osteoblast differentiation (via RUNX2, mTOR, YAP, BMP, PI3K/Akt) and chondrogenesis (via TGF-β/BMP signaling). E3 ligases also regulate osteoclast differentiation through pathways such as NF-κB, RANKL, and TRAF6. In addition, Parkin contributes to bone homeostasis by regulating mitophagy.

Although E3 ubiquitin ligases are conventionally classified based on their structural features, their functional contributions to skeletal homeostasis become more apparent when analyzed within the framework of specific regulatory processes. The maintenance of skeletal equilibrium depends on the coordinated interplay between osteoblast-driven bone formation, osteoclast-mediated bone resorption, and cartilage matrix turnover. At the same time, effective tissue remodeling and repair also require the integration of signaling pathways, progenitor cell responses, and microenvironmental factors (Salhotra et al., 2020; Miljkovic et al., 2008). Within this context, E3 ubiquitin ligases can be considered process-level modulators that regulate osteoblast and osteoclast differentiation, chondrogenesis, and other mechanisms (Figure 2).

### 3.1 E3 ubiquitin ligases in osteoblast regulation

#### 3.1.1 Regulation of osteogenesis through RUNX2 stability and transcriptional control

A recurrent mechanism by which E3 ubiquitin ligases influence osteogenesis is the regulation of RUNX2 stability and transcriptional activity. RUNX2, as the master transcription factor governing osteogenic lineage commitment, integrates multiple upstream signals to direct osteoblast differentiation. Its turnover via ubiquitination thus constitutes a critical regulatory checkpoint in bone formation. Within this framework, various E3 ligases and E3-associated regulatory axes can exert either pro-osteogenic or anti-osteogenic effects by modulating RUNX2 abundance and functional activity.

Among RING-type ligases, the tripartite motif (TRIM) protein family, defined by an N-terminal RBCC domain, encompasses multiple subfamilies with diverse regulatory roles, many of which have been implicated in skeletal homeostasis (Chen et al., 2021). Specifically, TRIM16 promotes osteogenic differentiation by attenuating CHIP-mediated K48-linked ubiquitination of RUNX2, thereby stabilizing RUNX2 in human periodontal ligament stem cells (Zhao et al., 2020). This mechanism underscores a pro-osteogenic function for TRIM16 by preserving RUNX2-dependent transcriptional output.

Similarly, members of the Cullin family, particularly CUL4, one of the most evolutionarily conserved Cullin–RING ligases (CRLs), exhibit positive effects on RUNX2-mediated osteogenesis. CUL4 exists as two isoforms, CUL4A and CUL4B, and participates in chromatin regulation and skeletal homeostasis (Chen et al., 2023; Yu et al., 2023; Nakagawa and Nakagawa, 2025). In bone marrow–derived mesenchymal stem cells (BMSCs), CUL4B supports osteogenesis via the KLF4–RUNX2 axis, as its deficiency diminishes bone mechanical strength and impairs regeneration, likely due to dysregulated KLF4-mediated control of RUNX2 expression (Yu et al., 2023; Nakagawa and Nakagawa, 2025). Unlike ligases that directly destabilize RUNX2, CUL4B maintains a transcriptional environment conducive to osteogenic gene expression.

Conversely, certain E3-regulated pathways suppress osteogenesis by enhancing RUNX2 degradation. For example, lysine demethylase 5A (KDM5A) can relieve miR-495-mediated repression of the CRL substrate receptor SKP2, thereby increasing SKP2-dependent ubiquitination and proteasomal degradation of RUNX2. This reduces RUNX2 protein stability, suppresses osteogenic differentiation, and impairs recovery from osteoporosis (Li et al., 2025c). These findings highlight that CRL-mediated substrate recognition can function as an anti-osteogenic module when coupled to RUNX2 proteolysis.

HECT-type ligases provide an additional layer of RUNX2-centered regulation. Members of the WWP family, including WWP1, WWP2, and ITCH, recognize PPXY motifs on substrate proteins and are implicated in bone metabolism (Tsunoda et al., 2022). WWP1 and ITCH act as negative regulators of osteogenesis by promoting ubiquitin-dependent degradation of RUNX2 and JunB in BMSCs, reducing osteoblast activity and mineralization (Zhao et al., 2011; Liu et al., 2017). In contrast to the stabilizing effects of TRIM16 or the supportive role of CUL4B, these ligases accelerate the depletion of osteogenic transcription factors.

Beyond direct RUNX2 turnover, several HECT-type ligases influence osteogenesis by modulating BMP/SMAD-dependent transcriptional programs. The SMURF family, initially identified as negative regulators of BMP and TGF-β signaling, promotes ubiquitination and degradation of key signaling components, acting as important modulators of skeletal development and bone homeostasis (Tang et al., 2018). SMURF1 and SMURF2 predominantly exert anti-osteogenic effects, although their substrate preferences vary. Loss of SMURF2 reduces Smad1/5 ubiquitination, enhancing BMP2-induced Smad1/5/8 phosphorylation and osteogenic differentiation in BMSCs (Kushioka et al., 2020). SMURF1 primarily suppresses bone formation by targeting canonical substrates, including Smad1, RUNX2, and MEKK2, thus modulating BMP- and MAPK-related signaling, while SMURF2 mainly regulates Smad-dependent pathways and transcription factors such as JunB. Some evidence suggests partial overlap in substrate specificity, reflecting context-dependent functional redundancy within the SMURF family (Song et al., 2023; Fang et al., 2021).

During osteogenesis, SMURF2 also directly promotes RUNX2 ubiquitination and degradation, an effect enhanced by FOXO1, which transcriptionally upregulates SMURF2, thereby suppressing osteogenic differentiation in valvular interstitial cells (VICs). Conversely, Hakai interacts with RUNX2, competitively interfering with SMURF2-mediated ubiquitination, stabilizing RUNX2, and enhancing its transcriptional activity (Jiang C. et al., 2024; Upadhyay et al., 2024). SMURF2 thus exhibits a dual function: promoting osteoblast differentiation via BMP/SMAD signaling while simultaneously suppressing osteogenesis by degrading RUNX2 via FOXO1-mediated degradation. Collectively, these results indicate that SMURF ligases modulate osteogenesis via both direct and indirect regulation of RUNX2-centered transcriptional programs.

In addition, some E3 ligases influence osteogenesis indirectly by regulating transcriptional coactivators. For instance, the RBR-type ligase HERC3 suppresses osteogenic differentiation and bone regeneration by promoting ubiquitination and degradation of the nuclear receptor coactivator NCOA1, thereby indirectly restraining RUNX2 activity (Li et al., 2023).

Together, these findings illustrate the multifaceted roles of E3 ubiquitin ligases in controlling osteogenesis through RUNX2-centered mechanisms. By either stabilizing or targeting RUNX2 for degradation, E3 ligases modulate osteoblast differentiation, with effects contingent on the specific ligase and regulatory context. These insights underscore the importance of E3 ligase-mediated proteostasis in maintaining bone homeostasis and highlight their potential as therapeutic targets in bone diseases.

#### 3.1.2 Regulation of osteogenesis through key signaling pathways and protein quality control mechanisms

BMP/SMAD signaling is a central regulatory pathway in osteogenesis, and E3 ubiquitin ligases play pivotal roles in modulating the stability and activity of critical signaling components that govern osteoblast differentiation and bone formation. RNF4, a 190-amino acid homodimeric RING E3 ligase, belongs to the SUMO-targeted ubiquitin ligase (STUbL) subfamily, which specifically ubiquitinates poly-SUMOylated substrates (DiBello et al., 2016; Barroso-Gomila et al., 2023). Accumulating evidence indicates that RNF4 is a key regulator of osteogenic differentiation in human bone marrow–derived mesenchymal stem cells (hBMSCs). Mechanistically, RNF4 activates the RNF4–BMP6–RGMb signaling axis, enhancing osteogenic signaling and differentiation, and thereby promoting bone formation (Novak et al., 2022). Through this pathway, RNF4 contributes substantially to bone homeostasis.

The PI3K/Akt and mTOR pathways are integral to osteoblast differentiation and skeletal metabolism, and E3 ligases modulate these cascades by regulating the turnover of proteins essential for cell growth, survival, and differentiation. HUWE1, a large HECT-type E3 ligase, contains a C-terminal HECT catalytic domain, ubiquitin-associated (UBA) and ubiquitin-binding (UBM) motifs, and a Bcl-2 homology 3 (BH3) domain (Wenmaekers et al., 2021). HUWE1 mediates polyubiquitination and delays internalization and degradation of the β-platelet-derived growth factor receptor (β-PDGFR), thereby prolonging its presence at the plasma membrane. This stabilization sustains PI3K/Akt signaling and enhances osteogenic differentiation in bone marrow mesenchymal stem cells (BMSCs) (Pham et al., 2022). Consequently, HUWE1 serves a critical function in PI3K/Akt-mediated osteogenesis, promoting bone formation by supporting key signaling events required for cellular growth and differentiation.

Conversely, c-CBL, a critical regulator of receptor tyrosine kinase (RTK) signaling, facilitates ubiquitin-dependent degradation of activated RTKs such as EGFR, thereby limiting excessive signaling and uncontrolled cellular proliferation (Su et al., 2024; Vogt et al., 2023). Additional evidence indicates that c-CBL also mediates ubiquitin-dependent mTOR degradation, counteracting cortactin (CTTN)-driven mTOR activation and ultimately inhibiting osteoblast differentiation (Spevak et al., 2020; Yang et al., 2024). These findings underscore c-CBL’s capacity to regulate osteogenesis via the modulation of mTOR signaling negatively.

Autophagy and protein quality control are vital for sustaining osteoblast function. E3 ubiquitin ligases contribute to these processes by promoting the degradation of misfolded proteins and regulating autophagic flux, ensuring proper bone formation and skeletal homeostasis. HECW1, a HECT, C2, and WW domain-containing ligase, has been increasingly associated with bone metabolism, despite its initial characterization in tumorigenesis, cell-cycle regulation, and neurodevelopment (Lin et al., 2023). Within the HECT family, HECW1 mediates K27-linked ubiquitination of YAP, facilitating its autophagic degradation. This activity suppresses osteogenic differentiation of tendon-derived stem cells (TDSCs) and mitigates trauma-induced heterotopic ossification (Jiang H. et al., 2024). Therefore, HECW1 plays a crucial role in osteogenesis by modulating autophagic flux and controlling the turnover of key signaling proteins, maintaining the balance between bone formation and homeostasis.

In summary, E3 ubiquitin ligases orchestrate osteogenesis by regulating pivotal signaling pathways, including BMP/SMAD, PI3K/Akt, and mTOR, and by maintaining protein quality control mechanisms such as autophagy. Through regulating the stability and degradation of essential signaling molecules, these ligases are central to osteoblast differentiation, skeletal homeostasis, and the coordination of bone formation and resorption. In Table 1, we summarize these E3 ubiquitin ligases, their associated targets or regulatory axes, molecular mechanisms, and corresponding osteogenic effects.

**TABLE 1:** E3 ubiquitin ligases in osteogenesis: Mechanisms, targets, and osteogenic effects.

E3 ligase	Target/axis	Molecular mechanism	Osteogenic effect	References
TRIM16	RUNX2	Inhibits RUNX2 ubiquitination	Promotes osteogenesis	Zhao et al. (2020)
CUL4B	KLF4/RUNX2	Regulates RUNX2 transcriptional axis	Promotes osteogenesis	Yu et al. (2023)
CRL complex	SKP2/RUNX2	SKP2-dependent RUNX2 ubiquitination and degradation	Suppresses osteogenesis	Li et al. (2025c)
WWP1/ITCH	RUNX2/JunB	Induces transcription factor degradation	Suppresses osteogenesis	Zhao et al. (2011), Liu et al. (2017)
HERC3	NCOA1/RUNX2	Degrades NCOA1 to suppress RUNX2 activity	Suppresses osteogenesis	Li et al. (2023)
SMURF1	Smad1/RUNX2/MEKK2	Targets BMP/MAPK components for degradation	Suppresses osteogenesis	Song et al. (2023), Fang et al. (2021)
SMURF2	Smad1/5/RUNX2	Context-dependent ubiquitination of signaling factors	Dual role in osteogenesis	Kushioka et al. (2020), Jiang et al. (2024a)
RNF4	BMP6/RGMb	Activates BMP signaling	Promotes osteogenesis	Novak et al. (2022)
HUWE1	β-PDGFR/PI3K/Akt signaling	Sustains receptor signaling	Promotes osteogenesis	Pham et al. (2022)
c-CBL	mTOR	Promotes mTOR degradation	Suppresses osteogenesis	Spevak et al. (2020), Yang et al. (2024)
HECW1	YAP	Promotes autophagic degradation of YAP	Suppresses osteogenesis	Jiang et al. (2024b)

### 3.2 E3 ubiquitin ligases in osteoclast regulation

A recurring mechanism by which E3 ubiquitin ligases influence osteoclast differentiation is the regulation of TRAF6-dependent signaling downstream of RANKL stimulation. TRAF6, functioning as a central adaptor in the RANKL/RANK signaling cascade, integrates upstream signals to activate NF-κB and associated pathways that drive osteoclast differentiation and maturation. Consequently, multiple E3 ligases modulate osteoclast formation by altering TRAF6 ubiquitination or the ubiquitination of related signaling components, thereby exerting either inhibitory or stimulatory effects on bone resorption.

Among anti-osteoclastogenic regulators, c-CBL–mediated ubiquitination of TRAF6 establishes a negative feedback loop that attenuates TRAF6 responsiveness to RANKL and IFN-γ stimulation. This regulation diminishes NF-κB activity, suppresses osteoclast differentiation and maturation, and exerts anti-resorptive effects on bone metabolism (Jang et al., 2019; Deng et al., 2025). Likewise, CBL-b, a related family member, mediates TRAF6 ubiquitination, a process modulated by intraflagellar transport protein 80 (IFT80). IFT80 enhances CBL-b–dependent TRAF6 ubiquitination, thereby inhibiting RANKL/RANK signaling and negatively regulating osteoclast differentiation (Deepak et al., 2022). In the same axis, TRIM38 suppresses RANKL-induced osteoclast differentiation by promoting K48-linked ubiquitination of TRAF6 (Feng et al., 2024). Collectively, these findings indicate that facilitating TRAF6 ubiquitination constitutes a key mechanism by which distinct E3 ligases restrain osteoclastogenic signaling.

Additional control of this pathway occurs via E3-associated ubiquitin-editing mechanisms. ITCH associates with the deubiquitinating enzyme CYLD to promote TRAF6 deubiquitination, thereby limiting RANKL-induced NF-κB activation and reducing osteoclast formation (Zhang et al., 2013). Although mechanistically distinct from ligases that directly enhance TRAF6 ubiquitination, this observation reinforces the concept that modulating TRAF6 ubiquitin dynamics represents a critical checkpoint in osteoclast differentiation.

Beyond direct regulation of TRAF6, other E3-dependent pathways also suppress bone resorption. For instance, icariin treatment has been shown to reduce CUL3 expression and decrease RANKL-induced ubiquitination of nuclear factor erythroid 2–related factor 2 (Nrf2) in RAW264.7 cells, thereby stabilizing Nrf2, inhibiting bone resorption, and alleviating osteoporosis-related pathology (Si et al., 2024).

Conversely, certain E3 ligases support osteoclast differentiation and function. E6-associated protein (E6AP, also termed UBE3A), the prototypical HECT E3 ligase, was initially identified as a cellular partner of human papillomavirus (HPV) E6 protein (Wang Z. et al., 2024). In UBE3A-deficient RAW264.7 cells, RANKL-induced cell fusion is impaired, leading to defective multinucleated osteoclast formation and reduced bone resorption. These findings indicate a crucial role for UBE3A in osteoclast differentiation and activity (Cho et al., 2022). Similarly, transcriptomic analyses suggest that HECW2 may participate in osteoclast differentiation pathways, supporting a potential regulatory function in bone resorption (Su et al., 2020).

Overall, these studies demonstrate that E3 ubiquitin ligases regulate osteoclast differentiation predominantly via TRAF6-centered signaling and ubiquitin-dependent control of osteoclast maturation. By either restraining osteoclastogenic signaling or promoting osteoclast activity, these ligases regulate bone resorption, highlighting the essential role of E3-mediated ubiquitin regulation in skeletal homeostasis. We summarize E3 ubiquitin ligases, their targets or regulatory axes, molecular mechanisms, and effects on osteoclast differentiation in Table 2.

**TABLE 2:** E3 ubiquitin ligases in osteoclast regulation.

E3 ligase	Target/axis	Molecular mechanism	Osteogenic effect	References
c-CBL	TRAF6	Promotes TRAF6 ubiquitination to suppress RANKL signaling	Inhibits osteoclast differentiation	Jang et al. (2019), Deng et al. (2025)
CBL-b	TRAF6	Enhances CBL-b–dependent TRAF6 ubiquitination via IFT80	Inhibits osteoclast differentiation	Deepak et al. (2022)
TRIM38	TRAF6	Enhances K48-linked ubiquitination of TRAF6	Inhibits osteoclast differentiation	Feng et al. (2024)
CUL3	Nrf2	Reduces RANKL-induced Nrf2 ubiquitination	Inhibits osteoclast differentiation	Si et al. (2024)
ITCH	TRAF6	Promotes TRAF6 deubiquitination through CYLD	Inhibits osteoclast differentiation	Zhang et al. (2013)
HECW2	​	Potential regulation of osteoclast differentiation	Promotes osteoclast differentiation	Su et al. (2020)
UBE3A	RANKL signaling	Regulates osteoclast differentiation by modulating RANKL signaling	Required for osteoclast differentiation	Cho et al. (2022)

### 3.3 E3 ubiquitin ligases in bone remodeling, osteoimmune coupling, and mitochondrial quality control

#### 3.3.1 E3 ligases coordinate osteoblast–osteoclast coupling during bone remodeling

Bone remodeling is characterized by the close functional coupling between osteoblast-mediated bone formation and osteoclast-driven bone resorption. Rather than acting independently on these processes, E3 ubiquitin ligases coordinate both arms of remodeling, thereby influencing whether osteoblast–osteoclast interactions remain balanced or shift toward net bone gain or loss.

Within this context, TRIM27 exerts bidirectional regulatory effects on skeletal homeostasis by simultaneously suppressing osteoclast differentiation and promoting osteoblast activity, potentially through TAB2 ubiquitination and NF-κB signaling inhibition (Kim et al., 2025). This dual functionality exemplifies a coupling mechanism in which concurrent inhibition of resorption and stimulation of formation favor bone accrual.

Protein quality control–associated E3 ligases also contribute to maintaining remodeling balance. STIP1 homology and U-box–containing protein 1 (STUB1) encodes the C-terminal Hsp70-interacting protein (CHIP), a representative U-box-containing RING E3 ligase involved in proteostasis via ubiquitination-dependent degradation of misfolded or aberrant proteins (Nadel et al., 2024; Zhao L. et al., 2024). In bone, CHIP maintains homeostasis, as its deficiency leads to aberrant NF-κB activation, increased osteoclastogenesis, impaired osteoblast differentiation, and consequent reductions in bone mass and quality (Wang et al., 2020). These coordinated effects on both osteoblast and osteoclast compartments illustrate that disruption of CHIP-dependent proteostasis drives a coupled shift toward bone loss.

HERC (HECT and RCC1-like domain–containing) proteins, defined by a C-terminal HECT catalytic domain combined with one or more RCC1-like domains (RLDs), are categorized into large HERCs (HERC1 and HERC2) and small HERCs (HERC3–HERC6) based on size and domain organization (Pérez-Villegas et al., 2022; Hochrainer et al., 2005). HERC1, the most extensively studied member, demonstrates dual regulatory functions in bone homeostasis. Mechanistically, HERC1 modulates C-RAF stability and downstream ERK/p38 signaling, thereby influencing osteoblast differentiation; loss of HERC1 stabilizes C-RAF and activates ERK/p38 signaling, enhancing mesenchymal stem cell osteogenic potential and increasing markers such as RUNX2, SP7, and ALP (He et al., 2025). However, micro-CT analysis of adult HERC1-deficient mice revealed substantial reductions in bone mass, indicating that activation of osteogenic signaling alone is insufficient to preserve skeletal homeostasis. Further studies demonstrated that HERC1 deficiency elevates the RANKL/OPG ratio, suggesting additional dysregulation of osteoclast-related processes and confirming that uncoupled osteoclastogenic signaling can override bone-forming effects (Pedrazza et al., 2023).

Collectively, TRIM27, CHIP/STUB1, and HERC1 exemplify that E3 ubiquitin ligases regulate bone remodeling not merely by modulating individual osteogenic or osteoclastic pathways, but by orchestrating the functional coupling between these processes. By coordinating or disrupting this balance, E3 ligases determine whether remodeling remains homeostatic or progresses toward pathological bone loss.

#### 3.3.2 E3 ligases in osteoimmune coupling and inflammatory remodeling

The immune microenvironment exerts a profound influence on bone remodeling, with macrophage polarization and inflammatory signaling shaping osteogenic and osteoclastic responses. E3 ubiquitin ligases contribute to osteoimmune coupling by modulating immune cell function and linking inflammatory cues to remodeling outcomes.

TRIM25 participates in this regulatory network by promoting M2 macrophage polarization by ubiquitinating triggering receptor expressed on myeloid cells 1 (TREM1) (Zhang et al., 2024b). As M2 macrophages are typically associated with tissue repair and pro-regenerative environments, TRIM25-mediated ubiquitination may facilitate bone regeneration by establishing an immune milieu conducive to osteogenesis.

Similarly, CHIP/STUB1-dependent mechanisms modulate osteoimmune interactions by controlling macrophage polarization and downstream repair processes. Inhibition of CHIP-mediated ubiquitination of PPARγ enhances M2 polarization and promotes angiogenesis and bone regeneration, whereas reduced STUB1-dependent ubiquitination of NRF2 mitigates osteoarthritis progression and supports intrinsic bone repair (Qiu et al., 2022; Cao et al., 2023). These findings illustrate that modulation of ubiquitination-dependent immune signaling can shift the balance from inflammatory damage toward regenerative outcomes.

Additionally, RNF31 (HOIP), a core catalytic component of the linear ubiquitin chain assembly complex (LUBAC), has emerged as a critical mediator of immune and inflammatory signaling (Zhang et al., 2024c). HOIP interacts with SMURF1 and induces its linear ubiquitination, thereby suppressing the activity of SMURF1, a negative regulator of osteogenesis, and consequently promoting bone regeneration (Fu et al., 2024).

In summary, these studies underscore that E3 ubiquitin ligases regulate bone remodeling not only through direct effects on osteoblasts and osteoclasts but also by shaping the immune microenvironment. By regulating macrophage polarization and inflammatory signaling, E3 ligases determine whether remodeling proceeds toward regeneration or toward inflammation-driven bone loss.

#### 3.3.3 Mitochondrial quality control and autophagy-related E3 pathways in bone homeostasis

Mitochondrial quality control and autophagy are increasingly recognized as important regulators of bone homeostasis, particularly under conditions of metabolic stress and inflammation. In this context, certain E3 ubiquitin ligases contribute to bone remodeling by modulating mitophagy and cellular stress responses, thereby indirectly influencing osteogenic and osteoclastic processes.

Parkin, encoded by the PRKN gene, is a representative member of the RBR family and was initially identified as a causative gene for early-onset Parkinson’s disease (PD). In the PINK1/Parkin pathway, PTEN-induced kinase 1 (PINK1) phosphorylates Parkin at Ser65, leading to activation of its E3 ligase activity and subsequent ubiquitin chain assembly on mitochondrial outer membrane proteins. These ubiquitin modifications serve as signals for autophagy receptor recruitment and trigger mitophagy, thereby maintaining mitochondrial quality control (Martinez et al., 2024; Fogo et al., 2025).

Growing evidence suggests that PINK1/Parkin-dependent mitophagy plays a role in the regulation of bone-related processes. Under osteogenic conditions, Parkin alleviates mitochondrial dysfunction and oxidative stress induced by high-fat exposure through PINK1/Parkin-mediated mitophagy, thereby restoring osteogenic signaling and increasing the expression of markers such as RUNX2 and COL1A1 (Yang et al., 2025). In contrast, suppression of Parkin activity has been shown to attenuate fluorosis-induced excessive autophagy and mitochondrial apoptosis, thereby protecting osteoblasts and partially mitigating bone injury (Hu et al., 2024). In inflammatory bone loss models, activation of the AMPK/BNIP3/PINK1/Parkin signaling axis enhances mitophagy, reduces reactive oxygen species (ROS) accumulation and inflammasome activation, limits M1 macrophage polarization and osteoclast overactivation, and ultimately alleviates inflammation-associated bone loss (Wang W. et al., 2024).

Notably, most available studies primarily emphasize Parkin’s role in mitophagy rather than its direct E3 ligase activity in osteogenic or osteoclastic signaling pathways. Although Parkin has been reported to ubiquitinate mitochondrial outer membrane proteins such as VDAC and MFN2, generating K63-linked ubiquitin chains that function as scaffolds for autophagic signaling (Ordureau et al., 2018), direct mechanistic links between Parkin-mediated ubiquitination and bone-regenerative signaling remain limited. These observations suggest that, in the context of skeletal biology, Parkin is more consistently associated with mitochondrial quality control than with substrate-specific ubiquitination events directly governing bone cell fate.

In addition to Parkin, ARIH1 is currently the only RBR family member clearly implicated in bone regulation. ARIH1 promotes ubiquitin–proteasome–dependent degradation of Rubicon, an endogenous inhibitor of autophagy, thereby restoring autophagic activity and increasing the expression of autophagy-related proteins (Beclin1 and ATG5) as well as osteogenic markers (RUNX2 and OCN). Through these effects, ARIH1 enhances osteogenic differentiation of bone marrow mesenchymal stem cells (BMSCs) (Xu et al., 2025).

Overall, these findings indicate that E3 ubiquitin ligases can influence bone remodeling by regulating mitochondrial quality control and autophagy-related pathways. However, compared with other regulatory axes, the direct contribution of E3 ligase–mediated ubiquitination to bone cell signaling remains less well defined, highlighting an area that requires further mechanistic investigation. A summary of E3 ubiquitin ligases, their targets or regulatory axes, molecular mechanisms, and effects on bone remodeling, osteoimmune interactions, and mitochondrial quality control is provided in Table 3.

**TABLE 3:** E3 ligases in bone remodeling, osteoimmune coupling, and mitochondrial quality control.

E3 ligase	Target/axis	Molecular mechanism	Bone remodeling effect	References
TRIM27	TAB2/NF-κB	Potentially ubiquitinates TAB2 and inhibits NF-κB signaling	Promotes bone formation and limits bone resorption	Kim et al. (2025)
TRIM25	TREM1	Promotes M2 macrophage polarization via TREM1 ubiquitination	Immune microenvironment promoting bone regeneration	Zhang et al. (2024b)
CHIP (STUB1)	NF-κB	CHIP deficiency leads to aberrant NF-κB activation	Protects bone mass; deficiency causes bone loss	Wang et al. (2020)
CHIP (STUB1)	PPARγ	Inhibition of CHIP-mediated PPARγ ubiquitination	Promotes angiogenesis and bone regeneration	Qiu et al. (2022)
CHIP (STUB1)	NRF2	Reduced STUB1-dependent NRF2 ubiquitination	Attenuates osteoarthritis progression and supports intrinsic repair	Cao et al. (2023)
HERC1	C-RAF/ERK/p38; RANKL/OPG	Modulates C-RAF stability and ERK/p38 signaling; HERC1 deficiency elevates RANKL/OPG ratio	Required for remodeling balance; loss leads to bone loss	He et al. (2025)
Parkin	PINK1/Parkin mitophagy; AMPK/BNIP3/PINK1/Parkin axis; VDAC/MFN2	Primarily studied in mitophagy/oxidative stress control; reported to ubiquitinate VDAC and MFN2 to generate K63-linked chains	Context-dependent; implicated in osteogenic and inflammatory bone injury settings	Yang et al. (2025), Hu et al. (2024), Wang et al. (2024a), Ordureau et al. (2018)
ARIH1	Rubicon/autophagy	Promotes UPS-dependent Rubicon degradation to restore autophagy	Promotes osteogenic differentiation	Xu et al. (2025)
RNF31 (HOIP)	SMURF1	Dampen the E3-ligase activity of SMURF1 by disrupting the E2-E3 interaction	Promotes osteogenic differentiation	Zhang et al. (2024c), Fu et al. (2024)

### 3.4 E3 ubiquitin ligases in chondrogenesis and cartilage homeostasis

In chondrogenesis and cartilage homeostasis, E3 ubiquitin ligases regulate key pathways that maintain the balance between cartilage matrix synthesis and degradation. An important regulatory axis involves the fine-tuning of TGF-β/BMP signaling through negative feedback mechanisms (Hanada et al., 2001; Murphy et al., 2015). Members of the NEDD4 family, such as SMURF1 and SMURF2, are recruited by inhibitory SMADs to promote the ubiquitination and degradation of receptor-regulated SMADs or upstream signaling components, thereby controlling chondrocyte differentiation. In particular, SMURF1 maintains SMAD6 function and delays hypertrophic differentiation of primary articular chondrocytes *in vivo* (Horiki et al., 2004). Furthermore, SMURF1 targets and promotes the degradation of SMAD1, inhibiting the TGF-β signaling pathway and potentially acting as a negative regulator of chondrocyte proliferation and migration (Cheng et al., 2025). SMURF2 promotes hypertrophic differentiation and chondrocyte maturation during endochondral development by targeting TGF-β receptor–activated SMAD2 and SMAD3 for proteasomal degradation. However, sustained or excessive SMURF2 activity in mature tissues contributes to degenerative changes, leading to spontaneous osteoarthritis (OA) and accelerated age-related intervertebral disc (IVD) degeneration (Wu et al., 2008; Wu and Huang, 2017).

Another key mechanism involves mitochondrial quality control. The RBR family E3 ligase Parkin maintains cartilage homeostasis by promoting PINK1-dependent mitophagy and facilitating the clearance of damaged mitochondria. Activation of the PINK1/Parkin pathway alleviates oxidative stress, chondrocyte senescence, and ferroptosis, thereby preserving extracellular matrix homeostasis (Xie et al., 2025; Zhuang et al., 2024). Conversely, disruption of this pathway impairs mitochondrial clearance and accelerates cartilage degeneration. For example, IRF1/PARP12-mediated ISGylation of MFN1/2 interferes with Parkin-dependent mitophagy, while the SPP1-ITGa5/β1 axis inhibits the PINK1/Parkin pathway and promotes nucleus pulposus cell degeneration and pathological calcification (Deng et al., 2024; Gu et al., 2025).

Collectively, these findings indicate that E3 ligases regulate cartilage homeostasis by coordinating differentiation signaling, mitochondrial quality control, and regenerative responses. Their disorders contribute to cartilage degeneration and osteoarthritis. In Table 4, we summarize E3 ubiquitin ligases, their target substrates, regulatory processes, and effects on chondrogenesis and cartilage homeostasis.

**TABLE 4:** E3 ubiquitin ligases in chondrogenesis and cartilage homeostasis.

E3 ligase	Target substrates	Regulatory process	Effect on cartilage	References
SMURF1	SMAD1/6	TGF-β/BMP signaling	Degenerative	Horiki et al. (2004), Cheng et al. (2025)
SMURF2	SMAD2/3	TGF-β/BMP signaling	Dual role	Wu et al. (2008), Wu and Huang (2017)
Parkin	PINK1/Parkin pathway components	Mitophagy	Protective	Xie et al. (2025), Zhuang et al. (2024), Gu et al. (2025), Deng et al. (2024)

**FIGURE 3:**
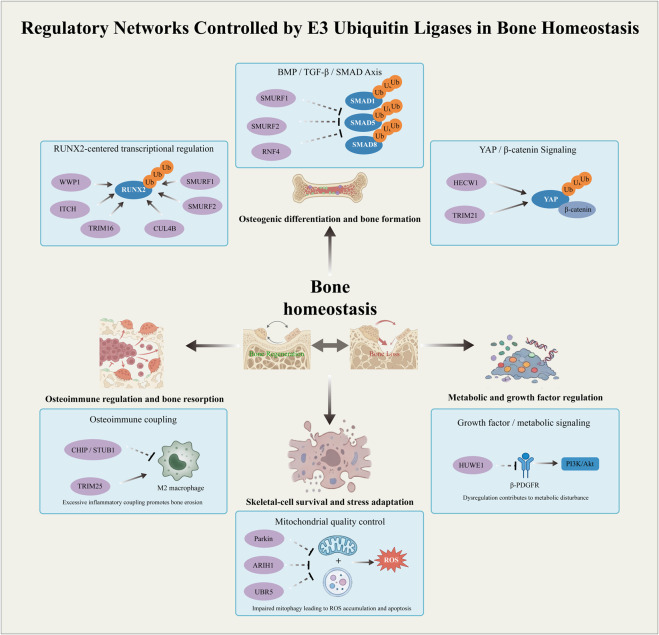
Regulatory networks controlled by E3 ubiquitin ligases in bone homeostasis: E3 ubiquitin ligases coordinate multiple processes to regulate bone homeostasis: osteogenic differentiation and bone formation, osteoimmune regulation and bone resorption, skeletal-cell survival and stress adaptation, and metabolic and growth factor regulation. In osteogenic and chondrogenic contexts, E3 ligases regulate differentiation through RUNX2-centered transcriptional control (including RUNX2, mTOR, Smad1/5, and MEKK2), BMP/TGF-β/SMAD signaling, and YAP/β-catenin signaling. In parallel, E3 ligases influence osteoimmune coupling, where factors such as CHIP/STUB1 and TRIM25 regulate macrophage polarization, and excessive inflammatory coupling promotes bone resorption. In addition, E3 ligases participate in metabolic and growth factor signaling, such as HUWE1-mediated regulation of β-PDGFR–PI3K/Akt signaling, linking metabolic disturbance to impaired bone homeostasis. They also govern mitochondrial quality control, in which ligases, including Parkin, ARIH1, and UBR5, regulate mitophagy and reactive oxygen species (ROS) accumulation, thereby affecting skeletal cell survival and stress adaptation.

In summary, E3 ubiquitin ligases regulate bone and cartilage homeostasis through coordinated control of osteoblast differentiation, osteoclast activity, osteoblast–osteoclast coupling, osteoimmune coupling, and mitochondrial quality control. These regulatory processes form multiple interconnected networks, including osteogenic differentiation and bone formation, osteoimmune regulation and bone resorption, skeletal-cell survival and stress adaptation, and metabolic and growth factor regulation (Figure 3). The dysregulation of these networks disrupts bone homeostasis and contributes to pathological skeletal remodeling.

## 4 E3 ubiquitin ligases in bone-related diseases

While previous sections have described the involvement of E3 ubiquitin ligases in regulating osteoblast and osteoclast differentiation, as well as processes including functional coupling, osteoimmune interactions, and mitochondrial quality control, growing evidence indicates that disruption of these mechanisms contributes to skeletal pathologies. Dysregulation of E3 ligase–mediated ubiquitination can compromise bone formation, increase bone resorption, and disturb overall tissue homeostasis. Elucidating these pathological alterations provides critical insights into the molecular underpinnings of bone disorders.

### 4.1 E3 ligases in osteoporosis and bone loss–related disorders

Altered activity of E3 ubiquitin ligases that govern osteogenic signaling represents a fundamental mechanism underlying bone loss–related diseases. As outlined in Section 3, osteoblast differentiation is tightly orchestrated by E3 ligase–mediated regulation of RUNX2 stability, key signaling pathways, and protein quality control. Perturbation of these regulatory axes diminishes osteogenic capacity and shifts the balance toward bone loss.

In osteoporosis, aberrant regulation of multiple E3 ligases has been associated with reduced bone formation and increased bone resorption. For example, TRIM21 levels are elevated in osteoporotic patients, and its depletion activates the YAP1/β-catenin pathway, resulting in decreased bone loss (Liu et al., 2023). Similarly, upregulation of CUL4A in ovariectomized mouse models promotes osteoclast differentiation and exacerbates bone loss, demonstrating how dysregulated ubiquitination disrupts the equilibrium between bone formation and resorption (Chen et al., 2023). Pharmacological modulation of SMURF-mediated ubiquitination enhances osteogenesis while suppressing osteoclast activity, mitigating bone loss in postmenopausal osteoporosis models (Fang et al., 2021). Consistent with these findings, icariin-mediated suppression of CUL3 stabilizes NRF2, inhibits osteoclast-related processes, and improves osteoporotic phenotypes (Si et al., 2024).

Beyond classical regulatory pathways, emerging evidence indicates that E3 ligase–associated noncoding RNAs contribute to osteogenic dysfunction in osteoporosis. Elevated circular RNA derived from HECW2 (circ_HECW2) correlates with increased osteoblast apoptosis, further linking dysregulated ubiquitin signaling to impaired bone formation (Zhang C. et al., 2024). E3 ligases also participate in inflammatory bone loss; for instance, TRIM14 attenuates NF-κB signaling by reducing p100 and p52 levels, alleviating bone loss in chronic periodontitis while preserving osteogenic potential (Zhang et al., 2022).

Mechanical unloading–induced bone loss represents another context of impaired osteogenic regulation. Upregulation of lncRNA Ubr5 under simulated microgravity conditions contributes to weightless bone loss and impairs proliferation and osteogenic differentiation of bone marrow mesenchymal stem cells (Wang et al., 2025). These findings suggest that E3 ligase–dependent regulatory networks are sensitive to biomechanical cues and may mediate bone loss under altered physical environments.

In addition to disease progression, dysregulation of osteogenic E3 ligases impacts bone repair. Dysregulated WW-domain-containing E3 ubiquitin protein ligase 1 (WWP1) contributes to age-related delays in fracture healing, as reduced miR-142-5p expression in aged callus is associated with impaired osteoblast activity. Targeting the miR-142-5p/WWP1 axis, however, can improve bone regeneration (Tu et al., 2017). These observations indicate that disruption of E3 ligase–mediated osteogenic programs not only drives bone loss but also compromises regenerative capacity.

E3 ligases also play roles in therapy-related skeletal complications. HERC4 has been implicated in bone metabolism through genetic association studies, in which variants at the HERC4 locus (rs3758392) were significantly associated with susceptibility to medication-related osteonecrosis of the jaw (MRONJ) (Yang et al., 2018; Wan et al., 2020). This evidence supports a role for HERC4 in osteoblast function and bone remodeling, suggesting that dysregulation of E3 ligase pathways may contribute to adverse skeletal outcomes under clinical interventions.

Collectively, these studies highlight that E3 ubiquitin ligases that control osteogenic pathways are central to multiple bone-loss–related conditions. Through modulation of RUNX2-centered transcription, signaling cascades, and protein stability regulation, dysregulation of these pathways results in diminished bone formation, increased resorption, and impaired skeletal repair.

### 4.2 E3 ligases in degenerative skeletal disorders

Dysregulation of E3 ubiquitin ligases involved in bone remodeling coordination represents a key mechanism underlying degenerative skeletal disorders. As discussed in Section 3, E3 ligases not only regulate osteoblast and osteoclast differentiation independently but also coordinate their functional coupling and maintain extracellular matrix homeostasis. Disruption of these integrative regulatory processes can lead to imbalanced remodeling and progressive tissue degeneration.

One prominent example is the HECT-type E3 ligase UBR5, which has been implicated in both bone remodeling and cartilage homeostasis. UBR5 is a structurally complex ligase containing multiple functional domains and plays an important role in maintaining skeletal integrity (Wang F. et al., 2023; Wang et al., 2025). Dysregulation of UBR5 has been associated with degenerative and ectopic skeletal pathology, as loss of UBR5 function enhances Hedgehog signaling and drives articular cartilage degradation, heterotopic ossification, and joint degeneration, with similar correlations observed in human osteoarthritic tissues (Mellis et al., 2021). These findings indicate that disruption of E3 ligase–mediated signaling integration can impair bone and cartilage homeostasis, ultimately contributing to the progression of degenerative disease.

In addition to cartilage degeneration, E3 ligases also regulate matrix homeostasis in intervertebral disc degeneration. Activation of CRL4 under conditions such as vitamin D deficiency promotes the degradation of nuclear receptor corepressor 2 (NCoR2), leading to enhanced extracellular matrix breakdown, impaired tissue repair, and accelerated degenerative changes (Li X. et al., 2025). Although this mechanism has primarily been characterized in intervertebral disc pathology, it highlights a broader role for E3 ligase–mediated ubiquitination in controlling matrix stability and tissue remodeling across skeletal compartments.

Collectively, these findings indicate that E3 ubiquitin ligases play essential roles in maintaining the balance of bone remodeling and matrix homeostasis. Their dysregulation disrupts osteoblast–osteoclast coupling, alters cartilage integrity, and accelerates degenerative processes, thereby contributing to the development of skeletal disorders characterized by progressive tissue breakdown. We summarize various bone-related diseases, the associated E3 ubiquitin ligases, their targets or regulatory axes, molecular mechanisms, and corresponding disease relevance in Table 5.

**TABLE 5:** E3 ubiquitin ligases in bone-related diseases.

Disease	E3 ligase	Target/axis	Molecular mechanism	Disease relevance	References
Osteoporosis	TRIM21	YAP1/β-catenin	TRIM21 depletion activates YAP1/β-catenin signaling	Reduces bone loss	Liu et al. (2023)
Osteoporosis	CUL4A	Osteoclast differentiation	Promotes osteoclastogenesis in the OVX model	Exacerbates bone loss	Chen et al. (2023)
Osteoporosis	SMURF	BMP/SMAD	Pharmacological modulation enhances osteogenesis and suppresses osteoclast activity	Attenuates bone loss	Fang et al. (2021)
Osteoporosis	CUL3	NRF2	Suppresses Nrf2 ubiquitination and stabilizes NRF2	Inhibits bone resorption and improves osteoporosis	Si et al. (2024)
Osteoporosis	HECW2 (circRNA)	Osteoblast apoptosis	circ_HECW2 is associated with increased apoptosis	Impairs bone formation	Zhang et al. (2024a)
Periodontitis	TRIM14	NF-κB	Reduces p100/p52 expression and NF-κB activity	Alleviates inflammatory bone loss	Zhang et al. (2022)
Microgravity bone loss	UBR5 (lncRNA)	Osteogenic differentiation	lncRNA Ubr5 upregulation impairs BMSC proliferation and osteogenesis	Promotes bone loss under unloading	Wang et al. (2025)
Fracture	WWP1	miR-142-5p axis	Dysregulation impairs osteoblast activity in aged callus	Delays fracture repair	Tu et al. (2017)
Cartilage degeneration/OA	UBR5	Hedgehog signaling	UBR5 loss enhances Hedgehog signaling	Drives cartilage degeneration and joint pathology	Mellis et al. (2021)
Intervertebral disc degeneration	CRL4	NCoR2	Promotes degradation of NCoR2 and ECM breakdown	Accelerates degenerative changes	Li et al. (2025a)
MRONJ	HERC4	Genetic association	SNP (rs3758392) linked to osteonecrosis risk	Associated with susceptibility to MRONJ	Yang et al. (2018), Wan et al. (2020)

## 5 Pharmacological targeting of E3 ligases and E3-dependent pathways in bone diseases

Bone diseases, including osteoporosis, osteoarthritis, and inflammatory bone loss, are significant clinical concerns. A variety of pharmacological treatments are currently available to reduce bone loss and support skeletal health. Osteoporosis, a common skeletal disorder characterized by reduced bone mass and increased fracture risk, is primarily managed with two classes of pharmacological agents. Antiresorptive drugs, such as Raloxifene, Alendronate, and Denosumab, slow down bone loss, whereas anabolic agents, including Teriparatide and Abaloparatide, promote new bone formation. All of these therapies have been approved by the Food and Drug Administration (FDA) (Yao et al., 2024; Tu et al., 2018).

In addition to osteoporosis, inflammatory bone loss disorders such as osteoarthritis and periodontitis remain significant clinical challenges. Current pharmacologic treatments for osteoarthritis include topical or oral nonsteroidal anti-inflammatory drugs (NSAIDs), topical capsaicin, and tramadol (Hochberg et al., 2012). Periodontitis is commonly managed with antibiotics such as amoxicillin and metronidazole (Arweiler et al., 2014). However, these therapies primarily alleviate symptoms or control infection, and do not specifically target the underlying inflammatory pathways driving bone damage.

Based on existing pharmacologic strategies for bone diseases, E3 ubiquitin ligases have recently emerged as attractive pharmacological targets due to their central role in specifying substrates within the UPS. Given their central role in specifying substrates within the UPS, E3 ubiquitin ligases have emerged as attractive pharmacological targets for treating skeletal disorders. Although direct E3-targeting therapeutics for bone diseases remain limited, increasing evidence indicates that E3-regulated pathways can be modulated via small molecules and targeted protein degradation technologies. Accordingly, E3 ligases are not only critical regulators of bone homeostasis but also represent potential druggable nodes for conditions such as osteoporosis, inflammatory bone loss, osteoarthritis, and bone repair (Peng et al., 2025; Gustin et al., 2006).

### 5.1 Direct E3-targeting drugs

A limited set of compounds acts directly on E3 ligases or their associated regulatory axes. For instance, TTP22 promotes the 20S proteasome-mediated degradation of TRAF6 by REGγ, thereby inhibiting osteoclastogenesis and alleviating osteoporosis (Du et al., 2024). Similarly, Morusinol restores FBXW7-dependent ubiquitination of PU.1 by disrupting the MSX2–PU.1 interaction, suppressing osteoclast fusion, reducing bone resorption, and enhancing angiogenesis-driven bone formation (Ma et al., 2025).

### 5.2 Drugs indirectly modulating E3-dependent pathways

Most pharmacological agents reported to date act indirectly by influencing E3-mediated ubiquitination or substrate stability. Several compounds exert pro-osteogenic effects by stabilizing key anabolic regulators. For example, Amlexanox prevents ubiquitin-mediated degradation of β-catenin, thereby promoting osteogenic signaling (He et al., 2024). Higenamine inhibits SMAD4 ubiquitination via the IQGAP1/SMAD4 axis, enhancing osteogenic differentiation and bone formation (Dong et al., 2023). Obeticholic acid suppresses Thoc6-mediated RUNX2 ubiquitination by activating FXR, stabilizing RUNX2, and promoting osteoblast-driven bone formation (Dong et al., 2025).

Indirect targeting of E3-dependent pathways can also mitigate pathological bone resorption and inflammation-induced bone loss. Coptisine chloride enhances TNFAIP3-mediated ubiquitination of NEK7, inhibiting inflammasome activation and inflammation-driven osteoclast activity (Zhou et al., 2024). Certain compounds operate through ubiquitination-related mitochondrial or stress-response mechanisms. For instance, PDZK1 supplementation and MitoQ preserve chondrocyte function via mitochondrial ubiquitination pathways and demonstrate skeletal protective effects (Shao et al., 2024). Pantethine suppresses K48-linked ubiquitination and degradation of SLC7A11, preventing ferroptosis in osteocytes and attenuating glucocorticoid-induced bone loss, though the responsible E3 ligase remains unidentified (Shi et al., 2025). Dimethyl fumarate inhibits TUFM ubiquitination and degradation, enhances mitophagy, and reduces inflammatory stress, supporting bone protection in inflammatory microenvironments (Chen et al., 2025).

### 5.3 Drug-based targeted protein degradation strategies

Targeted protein degradation strategies provide additional avenues for exploiting E3 ligases therapeutically. PROTACs are heterobifunctional molecules that recruit an E3 ligase to a specific target protein, leading to its ubiquitination and proteasomal degradation (Xiong et al., 2022). In skeletal research, MDM2-targeting PROTACs induce MDM2 degradation, thereby enhancing osteogenic differentiation and bone regeneration (Jeong et al., 2025). Similarly, CKIP-1-directed degraders offer potential therapeutic strategies for disuse osteoporosis, as CKIP-1 is a negative regulator of bone formation and can be eliminated via a VHL-dependent ubiquitin–proteasome pathway (Wei et al., 2024).

In summary, current pharmacological strategies targeting E3 ligases in bone diseases include direct modulation of E3 ligases or associated axes, indirect regulation of E3-dependent substrate turnover, and E3-based targeted protein degradation. Although direct E3-targeting therapeutics remain limited, these approaches collectively underscore the potential of manipulating E3-mediated protein homeostasis to enhance osteogenesis and suppress osteoclastogenesis or inflammatory bone loss. We summarize representative drug-based strategies targeting E3 ubiquitin ligases and E3-dependent pathways, including the corresponding E3 ligases or related axes, main actions, and effects on bone homeostasis, in Table 6.

**TABLE 6:** Representative drug-based strategies targeting E3 ligases and E3-dependent pathways in bone diseases.

Drug or therapeutic strategy	E3 ligase or related axis	Main action	Effect on bone homeostasis
TTP22	TRAF6	Promotes REGγ–20S dependent TRAF6 degradation	Inhibits osteoclastogenesis and alleviates osteoporotic bone loss (Du et al., 2024)
Morusinol	FBXW7–PU.1 axis	Restores FBXW7-dependent PU.1 ubiquitination	Suppresses osteoclast fusion, reduces bone resorption, and promotes coupled bone formation (Ma et al., 2025)
Amlexanox	β-catenin degradation pathway	Suppresses ubiquitin-mediated β-catenin degradation	Promotes osteogenic signaling and bone formation (He et al., 2024)
Higenamine	IQGAP1/SMAD4 axis	Inhibits SMAD4 ubiquitination	Promotes osteogenic differentiation and bone formation (Dong et al., 2023)
Obeticholic acid	Thoc6–RUNX2 axis	Suppresses Thoc6-mediated RUNX2 ubiquitination	Stabilizes RUNX2 and promotes osteoblast-driven bone formation (Dong et al., 2025)
Coptisine chloride	TNFAIP3–NEK7 axis	Enhances TNFAIP3-mediated NEK7 ubiquitination	Inhibits inflammatory bone resorption (Zhou et al., 2024)
PDZK1; MitoQ	Mitochondrial ubiquitination-related pathway	Preserves mitochondrial function and chondrocyte homeostasis	Preserves cartilage-related homeostasis and supports skeletal protection (Shao et al., 2024)
Pantethine	SLC7A11 ubiquitination pathway	Suppresses K48-linked SLC7A11 ubiquitination	Inhibits osteocytic ferroptosis and attenuates glucocorticoid-induced bone loss (Shi et al., 2025)
Dimethyl fumarate	TUFM ubiquitination pathway	Suppresses TUFM ubiquitination and degradation	Enhances mitophagy and reduces inflammatory stress in bone-related microenvironments (Chen et al., 2025)
MDM2-targeting PROTAC	MDM2	Induces MDM2 degradation	Promotes osteogenic differentiation and bone regeneration (Jeong et al., 2025)
CKIP-1-oriented PROTAC	CKIP-1; VHL	Enables VHL-dependent CKIP-1 degradation	Provides a potential osteoanabolic strategy for disuse osteoporosis (Wei et al., 2024)

## 6 Discussion and perspectives

This review reorganizes the roles of E3 ubiquitin ligases in skeletal biology from a process-oriented perspective, integrating their functions across osteoblast differentiation, osteoclast differentiation, cartilage homeostasis, and higher-order regulatory events such as osteoblast–osteoclast coupling, osteoimmune interactions, and mitochondrial quality control. Within this framework, E3 ligases emerge as key regulators of protein stability and signaling integration, coordinating cellular and microenvironmental processes underlying skeletal homeostasis. Notably, their functions frequently converge on critical regulatory nodes, including RUNX2 in osteogenesis and TRAF6-dependent signaling in osteoclast differentiation, while extending to broader network-level control of tissue remodeling.

Despite these advances, several challenges remain. The substrate specificity, context-dependent activity, and potential redundancy of many E3 ligases are still incompletely understood, and most studies have focused on individual targets rather than system-level ubiquitination networks. In addition, mechanistic insights are uneven across skeletal processes: while osteoblast and osteoclast regulation are relatively well characterized, emerging areas such as cartilage homeostasis, osteoimmune coupling, and mitochondrial quality control remain less defined. These limitations highlight the need for a more integrated understanding of E3 ligase–mediated regulation across different cellular and tissue contexts.

From a translational perspective, dysregulation of E3 ligase–dependent pathways contributes to a broad spectrum of skeletal diseases, including osteoporosis and degenerative disorders. E3-targeting therapeutics offer a promising strategy for conditions such as osteoarthritis and periodontitis, where current treatments lack pathway specificity. By modulating E3 ligases, it may be possible to achieve more precise control of pathological bone regeneration. Moreover, this approach could complement existing osteoporosis therapies, enhancing the balance between bone formation and resorption and supporting bone regeneration. Future studies should therefore focus on defining E3 ligase–substrate networks using proteomic approaches, integrating single-cell and spatial analyses to resolve microenvironmental regulation, and developing more physiologically relevant models that incorporate immune, metabolic, and mechanical cues. Such advances will be essential for translating mechanistic insights into targeted therapeutic strategies for skeletal disorders.

## Glossary

**UPS**: Ubiquitin–Proteasome System
**Ub**: Ubiquitin
**PTM**: Posttranslational Modification
**E1**: Ubiquitin-Activating Enzyme
**E2**: Ubiquitin-Conjugating Enzyme
**E3**: Ubiquitin Ligase
**DUB**: Deubiquitinating Enzyme
**RING**: Really Interesting New Gene
**HECT**: Homologous to the E6-AP Carboxyl Terminus
**RBR**: RING-Between-RING
**U-box**: U-box Domain
**CRL**: Cullin–RING ligase
**CUL**: Cullin
**SKP**: S-phase Kinase-Associated Protein
**RBX**: RING-Box protein
**CBL**: Casitas B-lineage Lymphoma
**RNF**: RING Finger Protein
**TRIM**: Tripartite Motif-Containing
**NEDD4**: Neural Precursor Cell Expressed Developmentally Downregulated 4
**SMURF**: SMAD Ubiquitination Regulatory Factor
**WWP**: WW Domain-Containing E3 Ubiquitin Protein Ligase
**HECW**: HECT, C2 and WW Domain-Containing
**HERC**: HECT and RCC1-Like Domain-Containing
**CHIP**: Carboxyl Terminus of Hsc70-Interacting Protein
**STUB1**: STIP1 Homology and U-Box Containing Protein 1
**ITCH**: Itchy E3 Ubiquitin Protein Ligase
**HUWE1**: HECT, UBA, and WWE Domain-Containing Protein 1
**UBE3A**: Ubiquitin Protein Ligase E3A
**UBR5**: Ubiquitin Protein Ligase E3 Component N-Recognin 5
**ARIH1**: Ariadne RBR E3 Ubiquitin Protein Ligase 1
**PARKIN**: Parkin RBR E3 Ubiquitin Protein Ligase
**RUNX2**: Runt-Related Transcription Factor 2
**SP7**: Sp7 Transcription Factor (Osterix)
**ALP**: Alkaline Phosphatase
**OCN**: Osteocalcin
**OPG**: Osteoprotegerin
**RANK**: Receptor Activator of Nuclear Factor κB
**RANKL**: Receptor Activator of Nuclear Factor κB Ligand
**BMSC**: Bone Marrow Mesenchymal Stem Cell
**RAW264.7**: Mouse Leukemic Monocyte Macrophage Cell Line
NF-κB: Nuclear Factor κB
**MAPK**: Mitogen-Activated Protein Kinase
**ERK**: Extracellular Signal-Regulated Kinase
**PI3K**: Phosphoinositide 3-Kinase
**AKT**: Protein Kinase B
**RTK**: Receptor Tyrosine Kinase
**EGFR**: Epidermal Growth Factor Receptor
**SMAD**: Small Mothers Against Decapentaplegic
**BMP**: Bone Morphogenetic Protein
**TGF-β**: Transforming Growth Factor Beta
**YAP**: Yes-Associated Protein
**PINK1**: PTEN-Induced Kinase 1
**ROS**: Reactive Oxygen Species
**AMPK**: AMP-Activated Protein Kinase
